# Extent of Knowledge about HIV and Its Determinants among Men in Bangladesh

**DOI:** 10.3389/fpubh.2016.00246

**Published:** 2016-11-03

**Authors:** Sanni Yaya, Ghose Bishwajit, Georges Danhoundo, Idé Seydou

**Affiliations:** ^1^Faculty of Social Sciences, School of International Development and Global Studies, University of Ottawa, Ottawa, ON, Canada; ^2^School of Medicine and Health Management, Tongji Medical College, Huazhong University of Science and Technology, Wuhan, China; ^3^Faculty of Health, York University, Toronto, ON, Canada; ^4^Faculty of Health Sciences, University of Ottawa, Ottawa, ON, Canada

**Keywords:** HIV knowledge, Bangladesh, demographic and health survey, education, global health, health communication

## Abstract

**Background:**

Bangladesh is currently a low human immunodeficiency virus (HIV) prevalent country. However, the risk factors are widespread and the number of at-risk population is also rising, which warrants special policy attention. The risks of transmission were shown to be correlated with the level of HIV knowledge of individuals. In this study, we aimed to explore the level and influencing factors of HIV knowledge among adult men in Bangladesh.

**Methodology:**

Data for the present study were collected from the sixth round of Bangladesh Demographic and Health Survey. Participants were 3305 men between 15 and 54 years of age regardless of HIV status. The primary outcome variable was the HIV knowledge score, which was calculated by responses to questions regarding general concepts and the mode of transmission of HIV. Association between the HIV knowledge score and the explanatory variables were analyzed by binary logistic regression methods.

**Result:**

The mean HIV knowledge score was 7.2 (SD 1.3). Results indicate that being an urban resident [*p* < 0.001; odds ratios (OR) = 0.56, 95% confidence intervals (CI) = 0.48–0.64], having secondary/higher educational level (*p* < 0.001 OR = 0.56, 95%CI = 0.48–0.64), reading newspaper [*p* = 0.006; OR = 0.76, 95%CI = 0.62–0.92], and communication with community health workers (CHWs) (*p* = 0.05; OR = 0.77, 95%CI = 0.60–10.00) were significantly associated with a high (equal or above mean value) HIV knowledge level.

**Conclusion:**

The level of HIV knowledge among Bangladeshi men is low. Leveraging HIV awareness programs targeting adult men to prevent future expansion of the epidemic should be a high priority. Revitalization and restructuring of the education sector and strengthening CHW’s engagement to improve knowledge about HIV transmission among men could generate beneficial returns for HIV prevention programs.

## Introduction

Globally, developing countries bear a disproportionate burden of the human immunodeficiency virus (HIV) cases. Despite extensive international efforts, the epidemic has survived almost four decades and continues to threaten millions of lives and undermine the development narratives in poor regions like South Asia and sub-Saharan Africa (1). Accounting for nearly a quarter of the global population, South Asia as a region so far has experienced a lower prevalence of HIV compared to the majority of other developing countries (2). Of the ~33.2 million global HIV cases (prevalence of 1.07%), nearly 95% of cases occur in developing countries, of which South Asia is estimated to account for about 2–3 million (2, 3). In South Asia, the prevalence ranges from as high as 0.3 in Nepal to as low as <0.1% in Bangladesh and Sri Lanka (3). The comparatively lower prevalence is attributed to the geographical detachment from the place of origin of the HIV virus, a low rate of in-migration, and HIV-testing regulations for long-term travelers and returning nationals (4).

Bangladesh has one of the lowest rates of HIV in the South Asian region (SAARC nations) (5); however, the risk of future expansion remains high, given the widespread prevalence of the risk factors, e.g., poverty (5), drug abuse (6), commercial sex (7), and an increasing number of cases in the neighboring countries (8). According to UNAIDS 2012, in Bangladesh, the rate of HIV has increased by about a quarter over the last decade (>25%) (4). In addition to that, the South Asian population shares an elevated risk of HIV morbidity due to high rates of tuberculosis (5). WHO ranks Bangladesh as one of the top 22 high TB burden countries in the world with an estimated prevalence of 411 (188–671)/100,000 population (9). People living with tuberculosis are reported to be more susceptible to HIV infection (10) and vice versa, and can account for one in four HIV mortality cases (2). As two major infectious diseases of poverty, the TB–HIV epidemic has been a major public health concern for the health-care system in Bangladesh (5).

Bangladesh is the third largest country in South Asia and sixth globally in terms of total population. Widespread poverty and malnutrition, low literacy, and high maternal and child mortality rates are the major public health issues in the country. Coupled with recent success in economic growth, the country has registered an appreciable achievement toward meeting the MDGs. However, HIV is an escalating concern given the high population density and poor health-care infrastructure. Since the detection of the first HIV case in 1986 (11), the country has made several programmatic efforts to curb the spread of HIV, such as provision of HIV testing and counseling (HTC) centers, drop-in-centers (DIC), as well as voluntary counseling and testing (VCT) under the national HIV intervention program. According to UNAIDS, Bangladesh is one of the few developing nations to launch early intervention policies to combat the epidemic (12) and has been on track to meet the Millennium Development Goal (MDG6) of halting HIV by 2015.

Several contributing factors to the upward trend of HIV in the country includes increase in intravenous drug users (IDUs), commercial sex workers (CSWs), and men who have sex with men (MSM) (4, 5). IDUs and CSWs tend to be un- or undereducated, which make them more challenging populations for effective health education (13). Moreover, low literacy rates and lack of public awareness regarding HIV constitute a serious obstacle for health-promotion programs. Understanding the factors that affect people’s knowledge and awareness of the epidemic especially with regard to the risk factors is of critical importance for designing comprehensive and contextual prevention frameworks. Another major barrier is the paucity of nationally representative data and evidence on HIV knowledge. To this end, we utilized the nationally representative Bangladesh Demographic and Health Survey (BDHS) data encompassing all six divisions to explore the factors that influence HIV knowledge and awareness among men in Bangladesh.

Previous studies have demonstrated that an individual’s health knowledge plays a dominant role in maintaining a healthy lifestyle (14) and length and quality of life (QoL) (15). The factors that influence health- and illness-related knowledge and utilization of the knowledge to adhere to healthy behavior (e.g., adoption/avoidance of risky lifestyle, personal health management) are usually connected to an individual’s socioeconomic and community conditions and their family environment (16–18). Among many factors, the impact of education is one of the most widely studied in the context of health-related behavior, which reported a positive effect of education on self-efficacy and healthy behavior (19, 20). The underlying link between education and health-related quality of life (HRQoL) is assumed to be the behavioral aspects that shape an individual’s perception of health and illness and the motivation for constant improvement (21).

A growing number of studies have reiterated the importance of behavioral interventions to achieve long-term public health goals especially in the context of the so-called lifestyle diseases or the non-communicable diseases (NCDs) (21). In contrast with NCDs, prevention of HIV infection is of critical importance as no cure is currently known. To this regard, behavioral approaches may prove effective particularly when efforts are made to tailor behavioral education and interventions at individuals and community levels (22). Studies have shown that increasing the knowledge of HIV can significantly reduce the risk of HIV transmission (15, 18, 23). The potential of behavioral intervention in curbing HIV-risk behaviors has been underscored by many researchers (21, 24, 25). A major challenge for behavioral intervention is that it cannot address the underlying condition of poverty, which is an important risk factor for poor health, low literacy, and overall socioeconomic well-being (26, 27). Poverty is commonly attributed as the culprit for which the easily preventable diseases account for the bulk of the global morbidity and mortality (28) and continues to thwart human development efforts (29). Based on these insights, the focus of the present study was to explore the factors of association of HIV knowledge with various sociodemographic (age, education, religion), individual (timing of sexual debut, number of wives), and economic (employment and perceived earning status) differentials among Bangladeshi men.

## Materials and Methods

### Details about the Survey and Sampling Procedure

DHS surveys are cross-sectional and nationally representative. BDHS 2011 was the sixth survey of its kind conducted in the country. DHS has been operating in the country since 1993 to provide quality information on adult men and women and under-5 children on a wide range of topics including demographic, socioeconomic, nutritional, family planning (FP), and health service use behavior. The aim is to help evaluate the health needs of the population and monitor the progress of public health programs (30). Fieldwork for BDHS 2011 survey lasted from 8th July through 27th December, 2011. The survey was implemented by the National Institute of Population Research and Training (NIPORT) and Mitra and Associates with technical assistance from ICF International (USA). Financial support was provided by the United States Agency for International Development (USAID), Bangladesh (30).

Sample was selected from all seven administrative divisions in the country: Barisal, Chittagong, Dhaka, Khulna, Rajshahi, Rangpur, and Sylhet and covered both urban and rural areas. The survey employed a two-stage cluster sampling strategy by using enumeration areas (EAs), which was a collection of about 120 households (22). Initially, 600 EAs were selected. In the second stage, on average, 30 households were selected systematically from each EA and finally 17,964 households were selected for interviews. However, interviews were successfully completed in 17,141 households (response rate of 98%). More details on the survey and sampling technique are available elsewhere (30, 31).

### Variables Selection and Measurement

The dependent variable in this study was the level of HIV Knowledge. A set of 10 questions pertinent to HIV knowledge was selected from the DHS questionnaire to which respondents could answer: yes/no/don’t know. Each correct and incorrect answer was respectively scored as “1” and “0.” Total score ranged from “0” to “’10.”

Independent variables in this study were categorized in the following way: age: 15–34 years and 35–54 years; type of residency: rural and urban; educational status: nil = 0 years of formal education, primary = 1–5 years of formal education, secondary/higher >6 years of formal education; self-reported income earning status: satisfactory, moderately satisfactory, and unsatisfactory; religion: Islam and other (Hinduism, Christianity, Buddhism); age at first sex: <15, 16–25, >25; number of wives: 1 and >1; had any sexually transmitted infection (STI) in last 12 months: yes/no; watch TV: yes/no; listen to radio: yes/no; read newspaper: yes/no; hearing about FP from a community event: yes/no; hearing about FP from a community health worker (CHW): yes/no; learned about FP from poster/billboard: yes/no.

### Data Analysis

Data analysis was performed using SPSS version 20.0. Sample characteristics were shown by descriptive statistics (weighed). The outcome variable (HIV score) was dichotomized into “high” and “low” with the latter encompassing those who scored below that of sample mean (<7) and high for those who scored equal or higher than that of sample mean (≥ 7). χ^2^ test of association was used to check for group differences (high vs. low score) in relation to the explanatory variables. Variables that showed significant association in the cross-tabulation were retained for final regression analysis. Association between HIV knowledge score and the explanatory variables was analyzed by binary logistic regression methods. Results were presented as odds ratios (OR) and corresponding confidence intervals (CI). *p*-value of <0.05 were considered statistically significant for all analyses.

### Ethical Approval

Informed consent was obtained from all respondents prior to interview. Interviewers explained that participation in the survey was voluntary. ICF International Institutional Review Board was the responsible body to approve the survey. Data sets are completely anonymous and distributed in the public domain of DHS without any identifiable information about the participants.

## Results

### Level of HIV Knowledge

Human immunodeficiency virus knowledge among the participants was assessed using a set of 10 questions on general awareness and knowledge about mode of transmission. Total score ranged from 0 to 10. Only 3% of the respondents could answer all the questions correctly. Less than one-tenth (8.2%) of all respondents got half or fewer of the answers correct and about one-third (32.9%) got correct answers for 70% of the questions.

The mean HIV knowledge score among men was 7.2 (SD 1.3). Score on general awareness (88%) was higher than that of knowledge about mode of transmission (78.4%). For all the questions, the percentage of men who knew correct answers was higher in urban areas (Table 1). All the respondents reported hearing about HIV and sexually transmitted diseases. About four-fifths of the respondents were of the opinion that using condoms and having one sex partner could reduce the risk of HIV infection. About one-fifth was of the opinion that a healthy looking person cannot have HIV.

**Table 1:** **Percentage of correct answers by questions**.

Questions	Percentage of correct answers (total sample)	Percentage of correct answers by region		*p*-Value
		Urban	Rural	
General awareness	88	90	88	
Ever heard of a sexually transmitted infection (STI)	100	100	100	–
Ever heard of AIDS	100	100	100	–
Reduce risk of getting HIV: have 1 sex partner only, who has no other partners	78.9	79.7	79.7	<0.0001
Reduce risk of getting HIV: always use condoms during sex	79.3	85.8	79.3	0.002
A healthy looking person can have HIV	82.8	83.5	82.4	<0.0001
Knowledge about mode of transmission	78	81	76	
Can get HIV through unsafe blood transfusions	94.1	94.2	92.6	0.281
Can get HIV by using unsterilized needle or syringe	92.7	94.3	91.6	0.002
Can get HIV by sharing food with person who has AIDS	57.3	63.8	53.2	0.512
Can get HIV from mosquito bites	58.2	61.7	55.1	0.0001
Can get HIV by witchcraft or supernatural means	90	91.7	89.0	<0.0001

*p-value calculated by chi-square tests*.

Concerning knowledge about the mode of transmission, over 90% of men were familiar with the possibility of HIV infection by using unsterilized needles or syringes and unsafe blood transfusions. One in ten respondents believed that HIV could be influenced by witchcraft or supernatural powers. The lowest correct response was for the questions of whether or not transmission can happen through mosquito bites (58.2) and sharing food with persons who have HIV (57.3).

### Baseline Characteristics of the Sample Population

Among the 3305 men included in the study, about 40% were aged between 15 and 34 years. One-third was of rural origin and about one-fifth had no formal education. About one-third of the participants had completed primary education. Forty six percent of participants had a secondary level of education or higher. Most participants were Muslim (87.2%), which is also the dominant faith in the country. Over a quarter of men reported having insufficient earnings to sustain their families. About 12% were satisfied and three-fifths were moderately satisfied. Almost all of the participants had only wife. The rate of early sexual debut (<15 years) was low (6.3%) and about one-third of the participants had experienced sex for the first time between the ages of 16 and 25. About half of the men reported reading newspapers. The percentage of utilization of TV and radio was 91.3 and 16.7, respectively. Only 2.5% of men reported having any STDs. Community clinic awareness was quite high (82.0), and 13.4% reported being visited by at least one FP personnel during the past 6 months.

Results of cross-tabulation are presented in Table 2 showing the comparison between participants in two groups based on their HIV knowledge scores in relation to the status of sociodemographic (age, type of residency, educational attainment), individual (number of wives, age at sex debut, STD status, reading newspaper, using TV and radio), and FP awareness (learning from community events, CHWs, and posters/billboards). Though participants’ ages were a significant factor, age at sex debut showed no statistically significant association with the level of HIV knowledge. Regarding media exposure, a higher proportion of men who reported watching TV and reading the newspaper had high knowledge of HIV.

**Table 2:** **Baseline characteristics of the study population, BDHS 2011**.

Variables	*N* (%)	Knowledge level		Chi	*p-*Value
		Low (41.4)	High (58.6)		
Age				**58.23**	<0.001*
15–34	1293 (39.1)	45.7	32.7		
35–54	2012 (60.9)	54.3	67.3		
Residency				**8.10**	0.002*
Urban	1291 (39.1)	36.6	41.4		
Rural	2014 (60.9)	63.4	58.6		
Employed				**0.094**	**0.434**
Yes	3252 (98.4)	98.5	98.3		
No	53 (1.6)	1.5	1.7		
Education				**26.97**	<0.001*
Nil	667 (20.2)	23.1	17.4		
Primary	1110 (33.6)	34.8	32.4		
Secondary/higher	1528 (46.2)	42.1	50.3		
Religion				**1.47**	**0.123**
Islam	2882 (87.2)	86.5	87.9		
Other	423 (12.8)	13.5	12.1		
Perceived earning status				**5.34**	**0.69**
Sufficient	394 (11.9)	10.9	12.9		
Moderately sufficient	2001 (60.5)	62.3	58.8		
Not sufficient	910 (27.5)	26.8	28.3		
Number of wives				**8.570**	**0.30**
1	3277 (99.2)	99.4	98.9		
>1	28 (0.8)	0.6	1.1		
Age at first sex				**1.47**	**0.12**
<15	208 (6.3)	5.8	6.2		
16–25	2427 (73.4)	51	66		
>25	670 (20.3)	43.2	27.8		
Read newspaper				**35**	<0.001*
Yes	1687 (51.0)	43.7	54.0		
No	1618 (49.0)	56.3	46.0		
Listens radio				**2.94**	**0.93**
Yes	553 (16.7)	15.6	17.8		
No	2752 (83.3)	84.4	82.2		
Watch TV				**3.97**	0.02*
Yes	3017 (91.3)	90.3	92.2		
No	288 (8.7)	9.7	7.8		
Had any STI in last 12 months				**14.27**	0.001*
Yes	84 (2.5)	1.6	3.5		
No	3221 (97.5)	98.4	96.5		
Heard about FP: from a community event				**2.5**	**0.62**
Yes	3055 (92.4)	6.8	8.3		
No	250 (7.6)	93.2	91.7		
Heard about FP poster/billboard				**1.62**	**0.115**
Yes	3075 (93.0)	6.4	7.5		
No	230 (7.0)	93.6	92.5		
Heard about FP from a community health worker				**4.91**	0.015*
Yes	2378 (72.0)	73.7	70.2		
No	927 (28.0)	26.3	29.8		

*FP, family planning*.

*Significant at p < 0.05.

### Factors Affecting Level of HIV Knowledge

The following variables were included in the regression model based on the association in the chi-square tests: age, type of residency, educational level, reading the newspaper, watching TV, having any STDs during the last year, and learning about FP from CHWs. Table 3 illustrates that odds of having high levels of HIV knowledge were 44% lower among rural men compared to their urban counterparts (*p* < 0.001). Though primary levels of education showed no significant association, the odds of having high HIV knowledge were 15 times higher among men who received secondary/higher education compared to those who had no formal education (*p* = 0.018, OR = 14.56, 95%CI = 10.59–130). Significant association was also found among those who read newspapers (*p* = 0.006, 95%CI = 0.62–0.92) and learned about FP from CHWs (*p* = 0.05, 95%CI = 0.60–10.00). Men who do not read newspapers and did not hear about FP from CHWs were, respectively, 25 and 23% less likely to score high on HIV knowledge. Though watching TV and having any STDs were found to be significant in the chi-square analysis, the association was lost in the logistic regression analysis.

**Table 3:** **Factors influencing HIV knowledge among men aging between 15 and 54 years in Bangladesh (BDHS 2011)**.

Variables	*B*	SE	*p*-Value	OR	95%CI
Age (15–34)^a^	–	–	–	–	–
35–54	0.118	0.075	ns	10.12	0.972–10.30
Residency (urban)^a^					
Rural	−0.575	0.073	<0.001*	0.56	0.48–0.64
Education (nil)^a^					
Primary	20.67	10.12	ns	60.31	0.72–540.9
Secondary/higher	10.84	10.10	0.018*	14.56	10.59–130
Read newspaper (yes)^a^					
No	−0.275	0.101	0.006*	0.759	0.62–0.92
TV (yes)^a^					
No	−0.178	0.128	ns	0.83	10.07
Had any STI in last 12 months (yes)^a^					
No	−0.01	0.08	ns	0.98	0.83–10.15
Heard about FP from a CHWs (yes)^a^					
No	−0.25	0.12	**0.05**	0.77	0.60–10.00

*OR, odds ratio; CHWs, community health workers*.

*^a^Reference category*.

*Significant at p < 0.05.

## Discussion and Conclusion

Bangladesh is currently a low HIV prevalent country compared to many of its Asian counterparts. However, the country cannot afford to take the situation lightly due to the high number of at-risk population (CSWs, IDUs). Moreover, given the current scenario of high population density coupled with widespread poverty and low literacy rates, the risk of future expansions of HIV remains high. The fact that there has been a net increase in total HIV cases over the last decades (3), albeit slowly, indicates the presence of flaws and inadequacies in the current HIV prevention efforts in the country. The etiology of HIV is multi-factorial and in order to be able to reverse its incidence, the health-care system must adopt more innovative and crosscutting strategies.

Insights from previous researches reveal that emphasis on raising public awareness regarding HIV is central to all HIV prevention strategies (21, 32). However, success will require significant financial and logistic investment to generate solid evidence through high-quality population-based studies. In this respect, our study bears a special significance as it opens the avenue for future researches regarding this issue in Bangladesh. Our findings suggest that the level of HIV knowledge among Bangladeshi men is low. Though all participants reported being aware of HIV, their understanding regarding modes of transmission is far from being adequate. Some three-fifths of the participants believed that HIV could be transmitted through mosquito bites and sharing food with an HIV positive person, and 10% believed in the association of supernatural forces with the disease.

Consistent with previous findings in India, our study demonstrated a positive association between HIV knowledge and the type of residency (21) and educational attainment (33, 34). Urban residents are more likely to be educated compared to their rural counterparts and tend to have better self-efficacy, awareness and show more adherence to healthy behaviors which are fundamental for HIV prevention. People with low educational levels are less likely to be aware of preventive measures, and, even when they are aware, the chance of non-compliance remains higher (35, 36). Compliance to regular condom use for instance, is higher among educated men and also more unlikely to have stigma associated with the disease (37). On the other hand, lack of knowledge and awareness is usually associated with stigmatization and poor health-care seeking which increases the risk of going undiagnosed (16, 38).

Our results support the fact that investing in the educational sector could translate to better prevention of HIV and can be regarded as an integral part of HIV policy making. Besides formal education, a counseling program and knowledge dissemination through religious affiliations have also shown to improve HIV knowledge and condom use among the youth (37). Religious institutions have a strong presence in the educational sector of Bangladesh (38) and can be engaged in enhancing HIV awareness as well.

The findings regarding the impact of newspaper reading on HIV knowledge further support the importance of education and knowledge dissemination. Still, a huge proportion of the population in the country lack connection to an electricity grid and rely on newspaper as the sole source of information. However, the effectiveness of newspaper in raising health awareness will depend on people’s level of trust on information channeled through this medium. Another important finding of our study was the impact of communication with FP workers on the level of HIV knowledge. Communication plays an instrumental role in knowledge production, sharing and growing positive health behaviors. In Bangladesh, CHWs are vital actors in delivering health messages in the remote and marginalized communities who are in high priority because of lack of access to basic health-care services. Thus, FP and outreach health workers can contribute considerably in HIV-knowledge building especially in the rural areas. When chosen from a similar environment, community-based workers can access people more reliably and help foster trust more effectively which is essential for convincing people to listen to them and to follow their advices. In a similar context, community engagement could also prove beneficial though its potential needs to be studied for making any concrete steps (13, 39, 40).

In conclusion, the level of HIV knowledge among men in Bangladesh remains inadequate. Leveraging the educational sector and making structural adjustments including compulsory primary education and incorporating adult education in the curriculum can pay off in the long run. Enhancing health communication through media and involving outreach workers in public awareness programs has the potential to improve HIV knowledge especially among men.

## Author Contributions

The study was conceptualized by SY and GB. Data analysis and interpretation of the results were done by SY, GB, GD, and IS. All the authors contributed to drafting and revision of the manuscript before approving the final version.

## Conflict of Interest Statement

The authors declare that the research was conducted in the absence of any commercial or financial relationships that could be construed as potential conflicts of interest.

## Abbreviations

BDHS, Bangladesh Demographic and Health Survey; CSWs, commercial sex workers; DIC, drop-in-centers; HRQoL, health-related quality of life; HTC, HIV testing and counseling; IDUs, intravenous drug users; MDG, millennium development goal; MSM, men who have sex with men; NCDs, non-communicable diseases; SAARC, South Asian Association for Regional Cooperation.
